# Scrotal epidermoid cysts penetrating the penile corpora cavernosa: Two case reports

**DOI:** 10.1002/iju5.12656

**Published:** 2023-10-10

**Authors:** Hiroshi Shimura, Atsuko Sato, Satoru Kira, Norifumi Sawada, Hiroyuki Satoh, Takahiko Mitsui

**Affiliations:** ^1^ Department of Urology University of Yamanashi Graduate School of Medical Sciences Yamanashi Japan; ^2^ Department of Pediatric Urology and Kidney Transplants Tokyo Metropolitan Children's Medical Center Tokyo Japan

**Keywords:** epidermoid cyst, intrascrotal tumor, pediatric tumor, penile corpora cavernosa, tumor resection

## Abstract

**Introduction:**

Epidermoid cysts are tumors and that rarely occur in intrascrotal extratesticular tissues. It is extremely rare for the tumors to penetrate the penile corpora cavernosa.

**Case presentation:**

We encountered a 4‐year‐old and a 6‐year‐old boy with intrascrotal tumors that penetrated the penile corpora cavernosa. Both the patients underwent tumor resection. In the former case, some of the tumor within the corpora cavernosa was left behind, while in the latter case, the tumor was completely resected. Pathological examination in both cases confirmed the diagnosis of epidermoid cysts.

**Conclusion:**

We should consider the possibility of epidermoid cysts in children presenting with intrascrotal tumors. Moreover, care should be taken when handling the corpora cavernosa during surgery.

**Abbreviation & Acronym**
MRI: Magnetic resonance imaging

**Keynote message**

We report two cases of epidermoid cysts. Both tumors occur in intrascrotal extratesticular tissues and penetrate the penile corpora cavernosa. This report informs the importance of considering the possibility of epidermoid cysts in children presenting with intrascrotal tumors and how to manage the tumor resection.

## Introduction

Epidermoid cysts are reportedly common in the anal region and ovaries.^1^ In urology, epidermoid cysts account for approximately 1% of all testicular tumors.^2^ The origin of epidermoid cysts in the ovaries or testes remains unknown; however, they are believed to be monolayer teratomas arising from germ cells.^2^ Epidermoid cysts rarely occur in intrascrotal extratesticular tissues^3^; the frequency of the occurrence is unknown.

We encountered two cases of intrascrotal but extratesticular epidermoid cysts that penetrated the penile corpora cavernosa. To the best of our knowledge, only one such case has been previously reported.^4^ Herein, we report two extremely rare cases of scrotal epidermoid cysts penetrating the penile corpora cavernosa and discuss the relevant literature.

## Case presentation

### Case 1

**Fig. 1:**
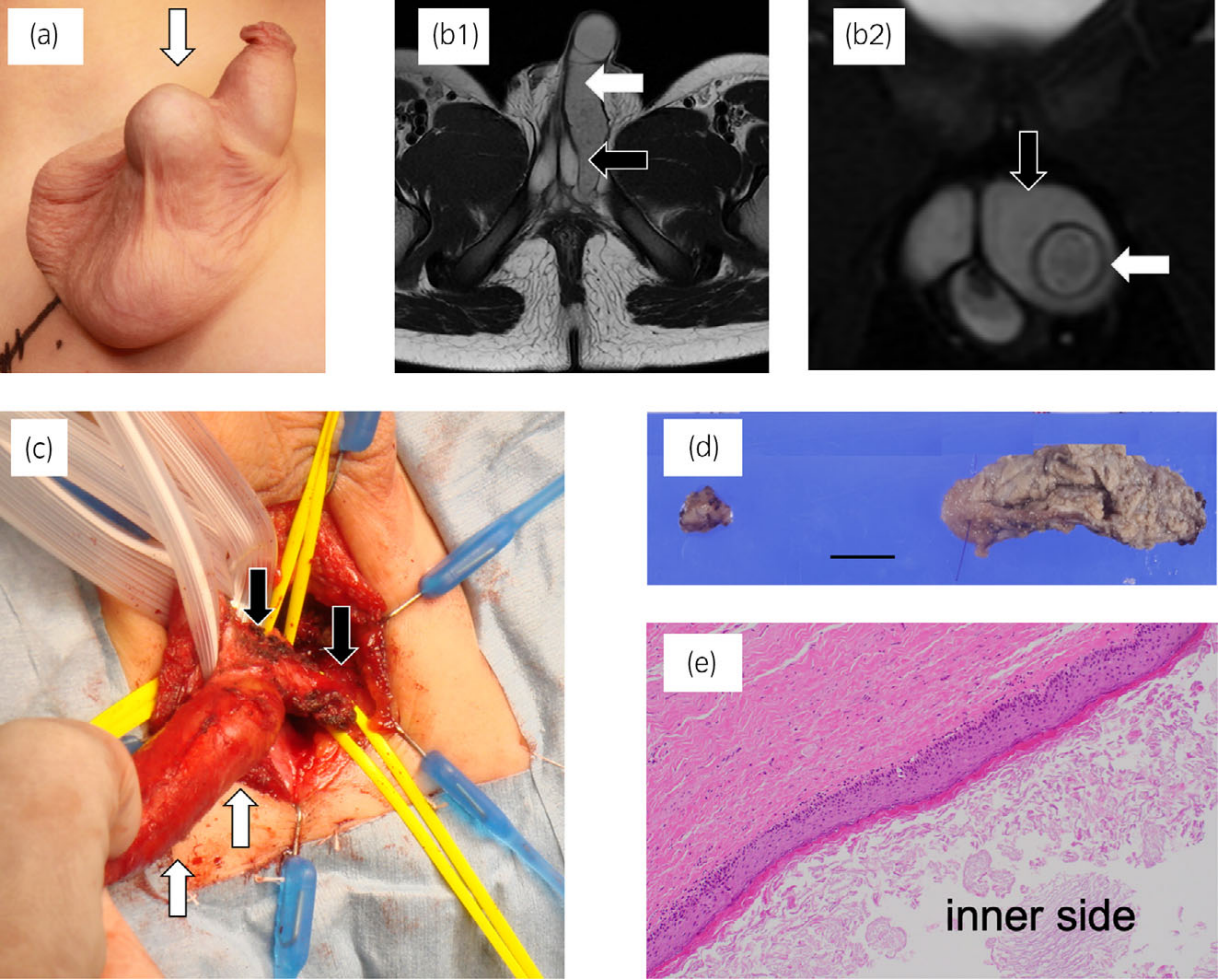
Case 1. (a) Appearance of the intrascrotal tumor: The masses were located in the midline of the scrotum and independent of the testes. (b) MRI: T2‐weighted axial (b1) and coronal (b2) images. The tubular mass extended from the scrotum to the pelvic floor with complete penetration of the penile corpora cavernosa. White arrow: tumor; black arrow: penile corpora cavernosa. (c) Operative findings: The thick tumor completely penetrated the left penile corpora cavernosa. White arrow: tumor; black arrow: penile corpora cavernosa. (d) Excised specimen: The peripheral (right) and central (left) parts of the tumor across the penile corpora cavernosa were excised separately. Black line: 10 mm. (e) Histopathological examination: The cyst walls were lined with stratified squamous epithelium without malignant changes. Hematoxylin and eosin (H&E) staining; magnification ×100.

A 4‐year‐old otherwise healthy boy presented to our hospital with a painless, rapidly enlarging tumor in the scrotum (Fig. 1a). The lesion was elastic, hard, smooth, and independent of the testes. The patient denied any previous trauma, inflammation, or urinary symptoms. Ultrasonography revealed an elongated, stick‐shaped cystic mass along the left side of the penis, extending into the pelvis. Doppler flow study showed no blood flow. MRI revealed a tubular, cystic non‐contrast‐enhancing lesion extending from the left scrotum, through the left penile corpora cavernosa, to the pelvic floor. The tumor had completely penetrated the corpora cavernosa (Fig. 1b1,b2). From the clinical course and imaging findings, our preoperative diagnosis was an epidermoid cyst as a benign cystic lesion.

After cystourethroscopy confirmed no connection with the urinary tract, the tumor was resected using a transperineal approach under general anesthesia. Once the tumor was identified and detached from the surrounding tissues, it was found to have penetrated the left penile corpora cavernosa (Fig. 1c). The peripheral and central sides of the tumor across the penile corpora cavernosa were excised (Fig. 1d). The tumor firmly strayed into the corpora cavernosa and left behind. The tunica albuginea of the penile corpora cavernosum was sutured at the resected edge; complete resection would have led to penile deformity.

Pathological examination revealed a cystic lesion lined with stratified squamous epithelium (Fig. 1e). The mild lymphocytic infiltration within the wall, confirmed the diagnosis of an epidermoid cyst. No postoperative recurrence or penile deformity has been observed to date. There seems to be no problem with erection.

### Case 2

**Fig. 2:**
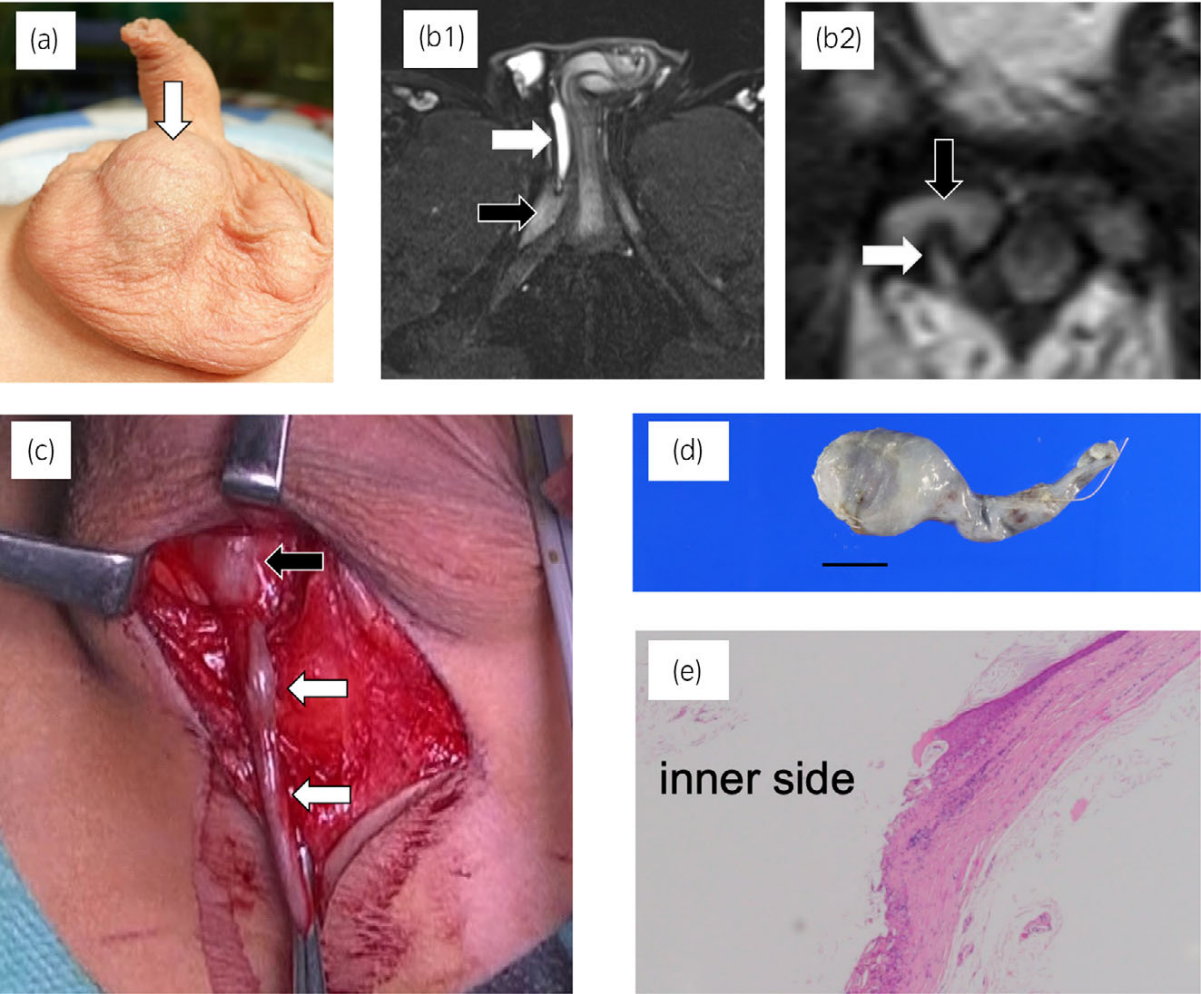
Case 2. (a) Appearance of the intrascrotal tumor: The masses were located in the midline of the scrotum and independent of the testes. (b) MRI: T2‐weighted axial (b1) and coronal (b2) images. The scrotal portion of the tumor was spherical and the dorsal portion was rod‐shaped with penetration of the right penile corpora cavernosa. White arrow: tumor; black arrow: penile corpora cavernosa. (c) Operative findings: The thin rod‐shaped part of the tumor penetrated the right penile corpora cavernosa. White arrow: tumor; black arrow: penile corpora cavernosa. (d) Excised specimen: The tumor consisted of spherical and rod‐shaped parts that were completely removed as one unit. Black line: 10 mm. (e) Histopathological examination: The cyst walls were lined with stratified squamous epithelium without malignant changes. Hematoxylin and eosin (H&E) staining; magnification ×100.

A 6‐year‐old boy presented to our hospital with a painless tumor in the scrotum, without any other symptoms (Fig. 2a). The well‐circumscribed mass was located at the midline of the scrotum and was independent of the testes. He had no history of trauma, inflammation, or urinary symptoms. MRI examination revealed a tumor with a cystic spherical ventral part and rod‐shaped dorsal part which penetrated the right penile corpora cavernosa (Fig. 2b1,b2). This case was also preoperatively diagnosed as an epidermoid cyst.

After cystourethroscopy, the tumor was resected as in Case 1, via a transperineal approach under general anesthesia. The rod‐shaped part of the tumor was first identified; the terminal end of the tumor entering the corpora cavernosum was bluntly dissected. Bleeding from the corpora cavernosum stopped spontaneously (Fig. 2c). Finally, the spherical body of the tumor was detached without difficulty and the tumor was completely resected (Fig. 2d).

Pathological examination confirmed the diagnosis of an epidermoid cyst, just as in Case 1 (Fig. 2e). No recurrence has been reported to date.

## Discussion

Epidermoid cyst is a dermatological tumor that is frequently encountered in the anal region and ovaries.^1^ The cyst wall has a normal structure with basal, spinous, and granular layers. Furthermore, an atheromatous keratinous substance is found in the area corresponding to the stratum corneum.^5^ Epidermoid cysts are often asymptomatic; rupture and secondary infection have been reported.^5^ Here, we report two rare cases of intrascrotal epidermoid cysts penetrating the penile corpora cavernosa.

Epidermoid cysts are benign, well‐circumscribed tumors of germ‐cell origin that represent approximately 1% of all testicular tumors. Reports of intrascrotal extratesticular epidermoid cysts are rare,^3^ often occurring in the midline of the perineum. Insufficient scrotal suturing or congenital stray epidermis are thought to cause epidermoid cysts. In our two cases and the only other reported case of epidermoid cyst penetrating the penile corpora cavernosa,^4^ no causative factor was identified. Furthermore, it is unlikely that a benign tumor, such as an epidermoid cyst, would grow through hard tissue such as the penile corpora cavernosa. Therefore, we propose that these epidermoid cysts which penetrate the penile corpora cavernosa develop during fetal life.

The tumor was resected in both patients in this study. The decision to excise or leave the part penetrating the penile corpora cavernosa was debated. If the tumor is left, there is concern about recurrence or malignant transformation. While malignant transformation of intracranial epidermoid cysts has been reported,^6^ malignancy itself is rare in scrotal epidermoid cysts,^7^ and if the biopsy or excisional specimen is benign, there will be no long‐term recurrence.^8^, ^9^ There have been no cases of postoperative recurrence of epidermoid cysts in the scrotum so far in the literature, and the risk of malignancy of epidermoid cysts may vary depending on the location of the cyst. In Case 1, the tumor within the corpora cavernosa was left behind to avoid penile deformity. If a complete resection were performed, the penile corpora cavernosa would be considerably damaged, and we thought that deformation of the penis could occur due to blood flow obstruction and suturing of the defect. From the excised specimen, the possibility of malignant transformation would be extremely low, and we believe that a follow‐up without additional resection is appropriate. Otherwise, in Case 2, the penetrating part could be easily pulled out. Thus, complete resection was possible. No recurrence or penile deformity was observed in either patient. Careful decision‐making regarding surgery is important in such cases after explaining to the patient the possibility of recurrence or penile deformity. Long‐term follow‐up is required in future studies.

## Conclusion

We encountered two cases of intrascrotal epidermoid cysts penetrating the penile corpora cavernosa. It is important to consider the possibility of epidermoid cysts in patients with scrotal tumors, which should be managed with tumor resection.

## Author contributions

Hiroshi Shimura: Conceptualization; data curation; investigation; methodology; validation; visualization; writing – original draft; writing – review and editing. Atsuko Sato: Validation. Satoru Kira: Validation. Norifumi Sawada: Validation. Hiroyuki Satoh: Validation. Takahiko Mitsui: Supervision; validation.

## Conflict of interest

The authors declare no conflict of interest.

## Approval of the research protocol by an Institutional Reviewer Board

Not Applicable.

## Informed consent

Informed consent was obtained from the patients' parents for publication of this case report and the accompanying images.

## Registry and the Registration No. of the study/trial

Not Applicable.
